# Global Spread of MCR-Producing *Salmonella enterica* Isolates

**DOI:** 10.3390/antibiotics11080998

**Published:** 2022-07-25

**Authors:** Zengfeng Zhang, Xiaorong Tian, Chunlei Shi

**Affiliations:** MOST-USDA Joint Research Center for Food Safety, School of Agriculture and Biology, and State Key Laboratory of Microbial Metabolism, Shanghai Jiao Tong University, Shanghai 200240, China; zhangzengfeng118@163.com (Z.Z.); tianxr@sjtu.edu.cn (X.T.)

**Keywords:** *Salmonella*, *mcr* genes, phylogenetic analysis

## Abstract

Colistin resistance in bacteria has become a significant threat to food safety and public health, and its development was mainly attributed to the plasmid-mediated *mcr* genes. This study aimed to determine the global prevalence and molecular characteristics of *mcr*-producing *Salmonella enterica* isolates. A total of 2279 *mcr*-producing *Salmonella* genomes were obtained from the public database, which were disseminated in 37 countries from five continents worldwide, including Asia, Europe, America, Australia, and Africa. Human samples (39.5%; 900/2279) were the predominant sources of *mcr*-producing *Salmonella* isolates, followed by foods (32.6%), animals (13.7%), and environment (4.4%). Furthermore, 80 *Salmonella* serotypes were identified, and Typhimurium and 1,4,[5],12:i:- were the predominant serotypes, accounting for 18.3% and 18.7%, respectively. Twenty *mcr* variants were identified, and the most common ones were *mcr-9.1* (65.2%) and *mcr-1.1* (24.4%). Carbapenems-resistance gene *bla*_NDM-1_ and tigecycline-resistance gene *tet*(X4) were identified in one isolate, respectively. Phylogenetic results indicated that *mcr*-producing *Salmonella* fell into nine lineages (Lineages I-IX), and *Salmonella* Typhimurium, 1,4,[5],12:i:- and 4,[5],12:i:- isolates from different countries were mixed in Lineages I, II and III, suggesting that international spread occurred. These findings underline further challenges for the spread of *Salmonella*-bearing *mcr* genes.

## 1. Introduction

Colistin was considered the last resort drug in the treatment for multidrug-resistant (MDR) and even carbapenem-resistant Gram-negative bacteria. Colistin-resistant bacteria have spread across Asia, Africa, Europe, North America, South America, and Oceania [1,2,3]. The colistin resistance in bacteria was previously reported to result from mutations in chromosomes [4]. Until 2015, the novel plasmid-borne colistin resistance gene *mcr-1* was firstly reported in an *Escherichia coli* isolate [5]. MCR-1 is a phosphoethanolamine (pEtN) transferase that is able to modify the phosphate groups in lipid A [5], the catalysis of which could be offered mainly by *mcr-1* from plasmids but also encoded by chromosome in the *Enterobacteriaceae* family [6].

Among countries with *mcr*-producing *Salmonella enterica* isolates, China was the most frequent one, and countries in Europe harbored a wide diversity of *mcr* variants [1]. The *mcr* gene was frequently identified in animal- and human-borne *Salmonella enterica* isolates [1]. Among more than 2600 *Salmonella* serotypes, *Salmonella* Typhimurium was the top serovar to carry the *mcr* genes [1]. Besides *Salmonella*, *Escherichia coli*, *Klebsiella Pneumoniae*, *Klebsiella oxytoca*, *Cronobacter sakazakii*, *Kluyvera ascorbata*, *Shigella sonnei*, *Citrobacter freundii*, *Citrobacter braakii*, *Raoultella ornithinolytica*, *Proteus mirabilis*, *Aeromonas*, *Moraxella,* and *Enterobacter* species were also found to be *mcr*-producing Gram-negative bacteria [3]. Furthermore, the *mcr* genes were widely distributed in various sample sources, including humans, animals, animal-origin foods, and environment [3]. 

Currently, a variety of *mcr* genes, including *mcr-1* to *-10,* have been reported in bacteria [6]. Furthermore, *mcr* genes were also found to co-exist with carbapenem resistance genes such as *bla*_VIM-1_, *bla*_NDM-5_, and *bla*_NDM-9_ [7,8,9]. Hence, the quick spread of plasmid-mediated *mcr* genes poses a significant threat to public health, requiring global surveillance.

Here, we characterized the global distribution of *mcr*-positive *Salmonella* using a data set of 2279 *Salmonella* genomes from humans, foods, animals, and environment. *Salmonella* serotypes, *mcr* variants, and phylogenomic characteristics were further analyzed.

## 2. Results

### 2.1. Widely Spread of mcr-Producing Salmonella Enterica in the Global and Sources Analysis

**Figure 1:**
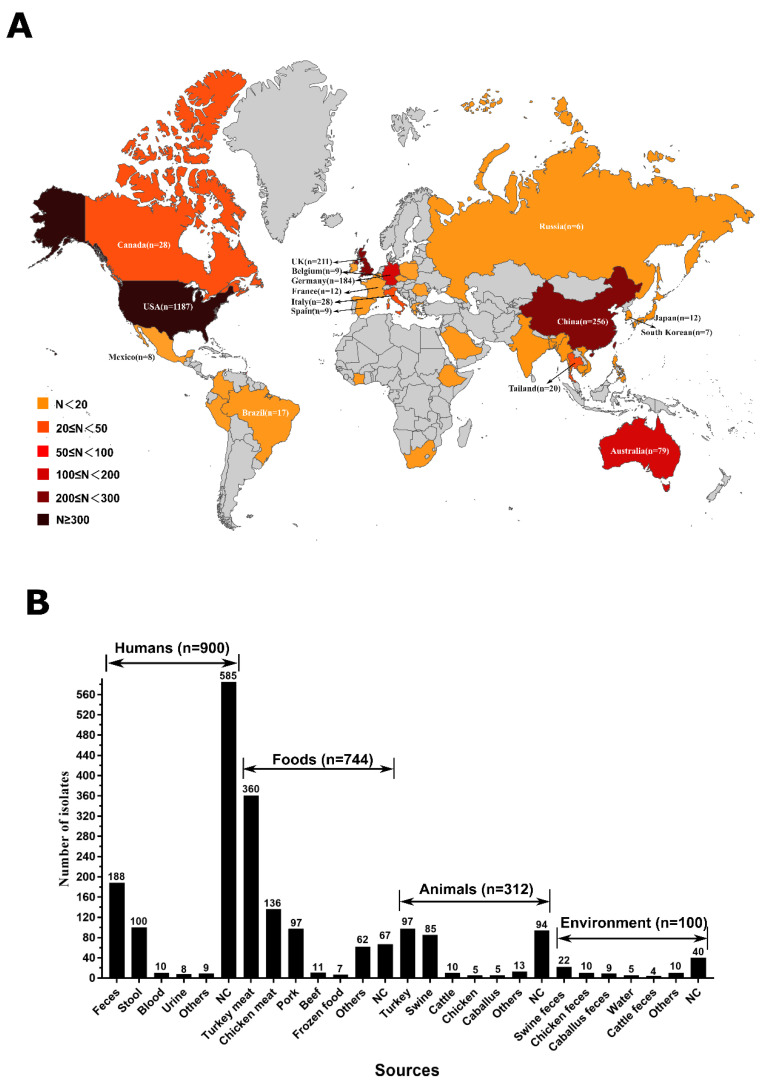
Global spread of MCR-producing *Salmonella enterica* isolates (**A**) and sources analysis (**B**).

Currently, a total of 2279 *mcr*-producing *Salmonella* genomes are available in the NCBI database. It was found that these *mcr*-producing *Salmonella* isolates had spread in 37 countries on five continents, including Asia, Europe, America, Australia, and Africa (Figure 1A). The top six countries are the United States (*n* = 1187), China (*n* = 256), United Kingdom (*n* = 211), Germany (*n* = 184), Australia (*n* = 79), and Canada (*n* = 28) (Figure 1A).

In this study, human samples (39.5%; 900/2279) were the predominant sources of *mcr*-producing *Salmonella* isolates (Figure 1B). It was further demonstrated that human samples were mainly composed of feces (*n* = 188), stool (*n* = 100), blood (*n* = 10), and urine (*n* = 8). A total of 744 (32.6%) isolates were recovered from foods. Turkey meats (*n* = 360) accounted for the largest portion, and then chicken meat (*n* = 136), pork (*n* = 97), beef (*n* = 11), and frozen foods (*n* = 7) (Figure 1B), which suggested that turkey meats were the main dissemination vehicle of *mcr*-producing *Salmonella* isolates. A total of 312 (13.7%) isolates were recovered from animals. Turkey (*n* = 97) accounted for the largest portion, and then swine (*n* = 85), cattle (*n* = 10), chicken (*n* = 5), and caballus (*n* = 5). In addition, 100 (4.4%) isolates were recovered from the environment, such as swine feces (*n* = 22), chicken feces (*n* = 10), caballus feces (*n* = 9), water (*n* = 5), and cattle feces (*n* = 4).

### 2.2. Typhimurium and 1,4,[5],12:i:- Were the Main Serotypes Carrying mcr Genes in Salmonella

**Figure 2:**
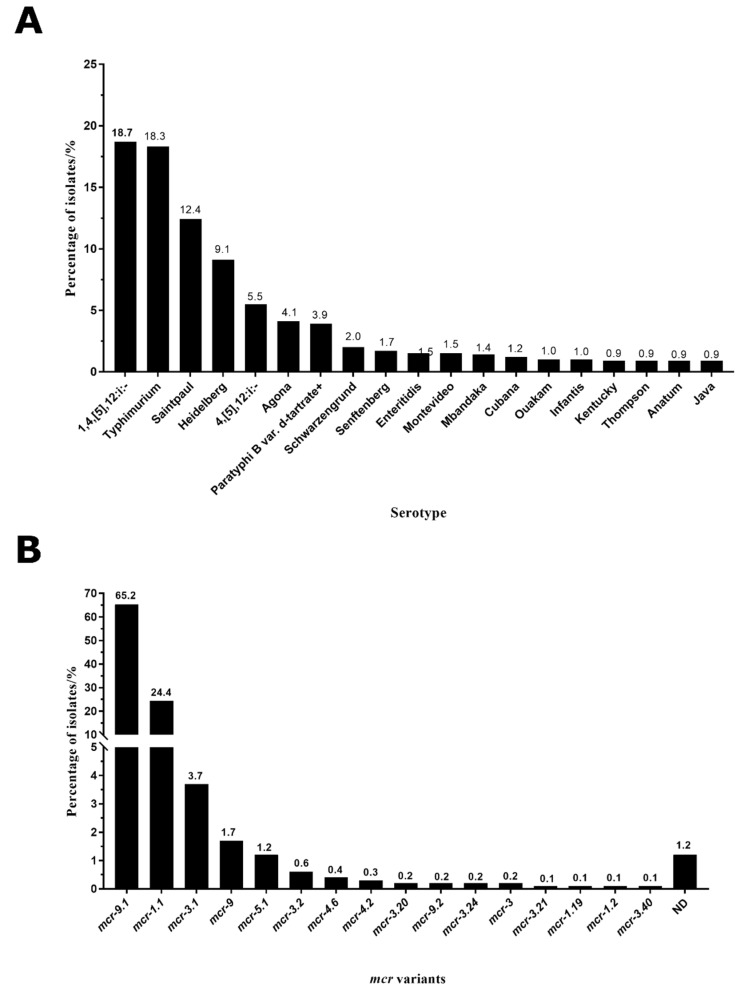
The distribution of the serotypes (**A**) and *mcr* variants (**B**) in MCR-producing *Salmonella*.

We collected and analyzed *Salmonella* serotypes in these 2279 isolates, and the missing serotypes information in some isolates was compensated by the predicted results from SeqSero 1.2 software (UGA College of Agricultural and Environmental Sciences food science, Athens, GA, USA). A total of 80 *Salmonella* serotypes were identified among these *Salmonella* isolates. Typhimurium and 1,4,[5],12:i:- were the predominant serotypes, accounting for 18.3% and 18.7%, respectively (Figure 2A). Other serotypes were Saintpaul (12.4%), Heidelberg (9.1%), 4,[5],12:i:- (5.5%), Agona (4.1%), Paratyphi B var. d-tartrate+ (3.9%), Schwarzengrund (2.0%), Senftenberg (1.7%), Enteritidis (1.5%), Montevideo (1.5%), Mbandaka (1.4%), Cubana (1.2%), Ouakam (1.0%), Infantis (1.0%), Kentucky (0.9%), Thompson (0.9%), Anatum (0.9%), and Java (0.9%) (Figure 2A).

**Figure 3:**
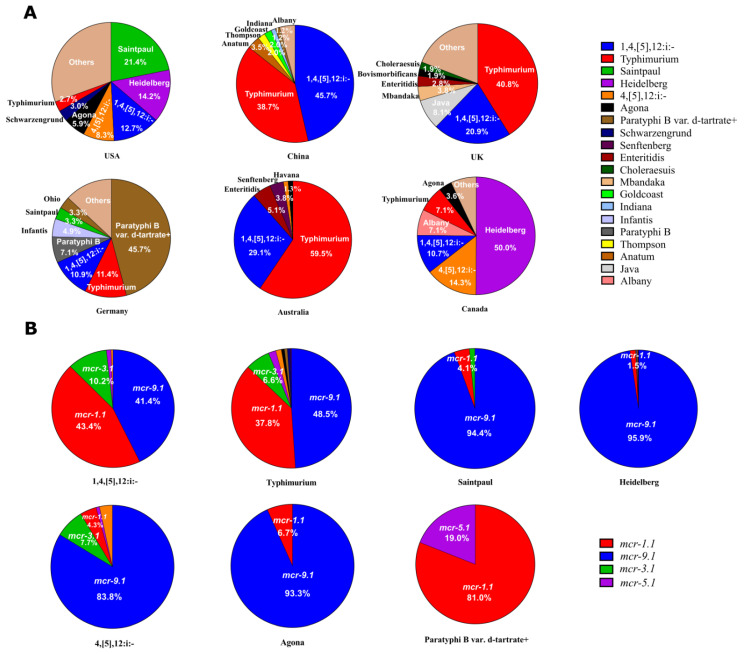
(**A**) The distribution of *mcr*-positive *Salmonella* serotypes in the USA, China, UK, Germany, Australia, and Canada. (**B**) The distribution of *mcr* variants in serotypes 1,4,[5],12:i:-, Typhimurium, Saintpaul, Heidelberg, 4,[5],12:i:-, Agona, and Paratyphi B var. d-tartrate+.

It was found that the prevalent *mcr*-producing *Salmonella* serotypes varied in different countries (Figure 3A). In the United States, the most common *Salmonella* serotype was Saintpaul (21.4%), followed by Heidelberg (14.2%), 1,4,[5],12:i:- (12.7%), 4,[5],12:i:- (8.3%), Agona (5.9%), Schwarzengrund (3.0%), and Typhimurium (2.7%) (Figure 3A). In China, the most frequently identified *Salmonella* serotype was 1,4,[5],12:i:- (45.7%), followed by Typhimurium (38.7%), Anatum (3.5%), Thompson (2.0%), Goldcoast (2.0%), Indiana (1.2%), and Albany (1.2%). In the United Kingdom, the most common *Salmonella* serotype was Typhimurium (40.8%), followed by 1,4,[5],12:i:- (20.9%), Java (8.1%), Mbandaka (3.8%), Enteritidis (2.8%), Bovismorbificans (1.9%), and Choleraesuis (1.9%). In the Germany, the most frequently identified *Salmonella* serotype was Paratyphi B var. d-tartrate+ (45.7%), followed by Typhimurium (11.4%), 1,4,[5],12:i:- (10.9%), Paratyphi B (7.1%), Infantis (4.9%), Saintpaul (3.3%), and Ohio (3.3%). In Australia, the most common *Salmonella* serotype was Typhimurium (59.5%), followed by 1,4,[5],12:i:- (29.1%), Enteritidis (5.1%), Senftenberg (3.8%), and Havana (1.3%). In Canada, the most frequently identified *Salmonella* serotype was Heidelberg (50.0%), followed by 4,[5],12:i:- (14.3%), 1,4,[5],12:i:- (10.7%), Albany (7.1%), Typhimurium (7.1%), and Agona (3.6%).

### 2.3. mcr-9.1 and mcr-1.1 Were the Dominant Variants in Salmonella

We identified the *mcr* variants from genomes through ResFinder 4.1 (Lyngby, Denmark). A total of 20 *mcr* variants were identified, and the most common variant was *mcr-9.1*, accounting for 65.2% (Figure 2B). Other *mcr* variants were *mcr-1.1* (24.4%), *mcr-3.1* (3.7%), *mcr-9* (1.7%), *mcr-5.1* (1.2%), *mcr-3.2* (0.6%), *mcr-4.6* (0.4%), *mcr-4.2* (0.3%), *mcr-3.20* (0.2%), *mcr-9.2* (0.2%), *mcr-3.24* (0.2%), *mcr-3* (0.2%), *mcr-3.21* (0.1%), *mcr-1.19* (0.1%), *mcr-1.2* (0.1%), and *mcr-3.40* (0.1%). The difference of *mcr* variant sequences could be found in Supplementary Materials. The partial or incomplete *mcr* genes could not be confirmed to a specific subtype, which were defined as not determined (ND) in Figure 2B, accounting for 1.2%.

Then, we analyzed the prevalence of *mcr* variants in the top seven *Salmonella* serotypes (1,4,[5],12:i:-, Typhimurium, Saintpaul, Heidelberg, 4,[5],12:i:-, Agona, and Paratyphi B var. d-tartrate+). It was found that *mcr-9.1* was the most common variant in these serotypes except for 1,4,[5],12:i:- (Figure 3B). Gene *mcr-1.1* (43.4%) was the most frequently identified variant in *Salmonella* 1,4,[5],12:i:-, followed by *mcr-9.1* (41.4%), and *mcr-3.1* (10.2%). Gene *mcr-9.1* (48.5%) was the most common variant in *Salmonella* Typhimurium, followed by *mcr-1.1* (37.8%), and *mcr-3.1* (6.6%). Gene *mcr-9.1* (94.4%) was the most frequently identified variant in *Salmonella* Saintpaul, and then *mcr-1.1* (4.1%). Gene *mcr-9.1* (95.9%) was the most common variant in *Salmonella* Heidelberg, and then *mcr-1.1* (1.5%). Gene *mcr-9.1* (83.8%) was the most frequently identified variant in *Salmonella* 4,[5],12:i:-, followed by *mcr-3.1* (7.7%), and *mcr-1.1* (4.3%). Gene *mcr-9.1* (93.3%) was the most common variant in *Salmonella* Agona, and then *mcr-1.1* (6.7%). Gene *mcr-1.1* (81.0%) was the most frequently identified variant in *Salmonella* Paratyphi B var. d-tartrate+, and then *mcr-5.1* (19.0%).

### 2.4. Antimicrobial Resistance (AMR) Genotypes in mcr-Producing Salmonella Isolates

A total of 68 acquired AMR genes were found in these *mcr*-producing *Salmonella* isolates (Table 1). Carbapenems-resistance gene *bla*_NDM-1_ was identified in one isolate. It was noted that a tigecycline-resistance gene *tet(*X4) was identified in one isolate. Furthermore, a total of 8 *bla*_CTX-M_ variants were identified, *bla*_CTX-M-14_ (6.0%) of which were the most common one, followed by *bla*_CTX-M-9_ (4.8%), *bla*_CTX-M-55_ (3.9%), *bla*_CTX-M-15_ (0.8%), *bla*_CTX-M-65_ (0.6%), *bla*_CTX-M-3_ (0.6%), *bla*_CTX-M-2_ (0.2%), and *bla*_CTX-M-130_ (0.1%). Gene *mph*(A) (5.0%) was the most common macrolides-resistance gene, then *erm*(B) (0.4%), *mef*(B) (0.3%), and *erm*(42) (0.2%). Fosfomycin-resistance gene *fos*A7 and *fos*A3 accounted for 14.3% and 6.0%, respectively. Gene *qnr*B2 (9.4%) was the most common plasmid-mediated quinolones-resistance (PMQR) gene, followed by *oqx*AB (7.9%), *qnr*S1 (7.0%), *qnr*B19 (2.6%), *qnr*B4 (1.4%), and *qnr*S2 (0.4%). Tetracycline-resistance genes *tet*(B) (48.2%), *tet*(A) (25.4%), *tet*(D) (16.1%), *tet*(M) (3.1%), and *tet*(C) (1.5%) were identified. Mutations in genes associated with AMR from genomes were also identified. It was found that *gyr*A_D87G (5.6%) was the most common mutation, followed by *gyr*A_D87N (4.3%), *gyr*A_S83Y (2.5%), *gyr*A_S83F (2.5%), *par*C_S80I (0.5%), and *par*C_S80R (0.2%). In addition, *gyr*A_G81C and *par*E_S458P were identified in one isolate, respectively.

### 2.5. Phylogenomic Analysis of mcr-Producing Salmonella

**Figure 4:**
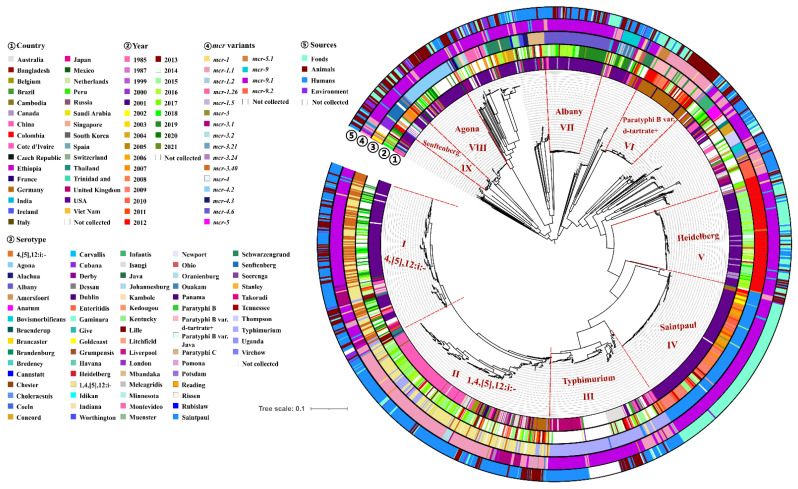
Phylogenetic tree of 2145 *Salmonella* genomes from different countries. Circles ①–⑤ denote countries, years, serotypes, *mcr* variants, and sources, respectively. The detailed information in circles ①–⑤ using various colors shown in the key.

To provide the evolutional characteristics of *mcr*-producing *Salmonella*, we compared 2145 genomes of different *Salmonella* serotypes from different countries. A total of 25,735 core SNPs were identified to construct a maximum likelihood tree (Figure 4). The evolutional strategy was based on serotypes. Currently, among 2145 isolates, the gene *mcr* (*mcr-5.1*) could be traced back to a *Salmonella* Typhimurium isolate in 1985, which was recovered from *Bos taurus* in Japan. It was found that Typhimurium and 1,4,[5],12:i:- as well as the close serotypes such as Saintpaul, Heidelberg and 4,[5],12:i:- were the main serotypes carrying *mcr* genes in *Salmonella* (Figure 4). The *Salmonella* Typhimurium, 1,4,[5],12:i:- and 4,[5],12:i:- isolates were distributed in Lineages I, II, and III. The phylogenetic tree showed that Lineages I and II were mixed clusters, suggesting their close genetic relationship. Lineage I was mainly composed of *Salmonella* 4,[5],12:i:- and 1,4,[5],12:i:- isolates. Lineage II was mainly composed of *Salmonella* 1,4,[5],12:i:- as well as some *Salmonella* 4,[5],12:i:- and *Salmonella* Typhimurium isolates. Lineage III was almost composed of *Salmonella* Typhimurium isolates. Furthermore, *Salmonella* Typhimurium isolates from the United Kingdom and Australia in Lineage III were divided into two sub-clades, respectively. It was found that most isolates of Lineage II were from China, but the base isolates were recovered from Germany, suggesting that *mcr*-producing *Salmonella* 1,4,[5],12:i:- isolates might be introduced into China from Germany. Similar results were also found in Lineage IV. Lineage IV was composed of *Salmonella* Saintpaul isolates from the USA, and their evolutionary path implied that a major introduction might occur in the USA from Germany and then nationwide spread. Indeed, the predominant serotype carrying *mcr* genes in Germany was *Salmonella* Paratyphi B var. d-tartrate+, which could be found in Lineage VI. We also found that isolates from Lineages I, II, V, VII, VIII, and IX were mostly isolated after 2013, but those from Lineages IV and VI were mainly isolated from 2002 to 2013. In addition, isolates from different sources shared a close genetic relationship, which suggested that clonal spread occurred. For example, most isolates of Lineage II were recovered from humans but also clustered with some isolates from animals. Similar results were also found in isolates of Lineages I and III.

## 3. Discussion

Colistin had been considered a therapeutic drug and feed additive for animals since the early 1980s and was approved for clinical treatment of human beings in 2017 in China [10,11]. In a previous study, *mcr-1*-producing *E. coli* isolates were able to transfer from animals to humans through the chicken production chain [12]. *E. coli*, *E. cloacae*, *Salmonella* spp., and *K. pneumonia* were reported to be the dominant *mcr-1*-producing bacteria members [1,5,11,13]. China’s government has banned colistin as a feed additive to prevent colistin resistance in clinics, and then the drug production decreased markedly from 27,170 tons in 2015 to 2,497 tons in 2018, and the corresponding sale also decreased from USD 71.5 million in 2015 to USD 8.0 million in 2018 in China [14]. Furthermore, the median relative abundance of *mcr-1* per 16S RNA in *E. coli* from animals and humans decreased significantly from 0.0009 in 2017 to 0.0002 in 2018 [14]. Therefore, the ban on colistin in animal farming causes a significant decrease in *mcr-1* of *E. coli* isolates from animals and humans.

The emergence of plasmid-borne *mcr* genes has attracted significant attention worldwide [3,5]. In a previous study about the prevalence of *mcr*-producing bacterial isolates, it was found that a significant increase occurred in 2009 [3]. The chicken-borne *mcr-1*-producing *E. coli* isolates increased from 5.2% in 2009 to 30.0% in 2014 [15]. In a retrospective survey from China, the earliest chicken-borne *mcr-1*-producing *E. coli* isolate was found in the 1980s, which corresponded to the start use of colistin in animal husbandry as a feed additive [15]. In this study, the earliest emergence of *mcr* was identified from a *Salmonella* Typhimurium isolate from *Bos taurus* in 1985 (Japan, *mcr-5.1*), and the earliest *mcr* gene from humans was identified in a *Salmonella* 1,4,[5],12:i:- isolate in 2001 (the USA, *mcr-9.1*). Up to 1 January 2022, a total of 2279 *mcr*-positive *Salmonella* genomes were available from the NCBI database and were widely spread in 37 countries. Therefore, it was necessary to monitor the prevalence of *mcr-1* in *Salmonella* to evaluate its burden on human health.

In this study, 80 *Salmonella* serotypes were identified to be carrying *mcr* genes, among which Typhimurium (18.3%) and 1,4,[5],12:i:- (18.7%) were the predominant ones. *S*. Typhimurium and 1,4,[5],12:i:- generally exhibited high-level prevalence and strong MDR features and spread worldwide [16]. Furthermore, the top three *mcr* variants in both Typhimurium and 1,4,[5],12:i:- were *mcr-1.1*, *mcr-9.1,* and *mcr-3.1*. Typhimurium and 1,4,[5],12:i:- were the predominant serotypes carrying *mcr* genes in China, the UK, and Australia. Furthermore, the phylogenetic relationship of *S*. Typhimurium and 1,4,[5],12:i:- isolates from these three countries was close, suggesting the possibility of the international spread among them. Unlike China, 1,4,[5],12:i:- (12.7%) was the third serotype carrying *mcr* genes in the USA, lower than Saintpaul (21.4%) and Heidelberg (14.2%). In Germany, Paratyphi B var. d-tartrate+ was the predominant serotype carrying *mcr* genes, and the top 2 *mcr* variants were *mcr-1.1* and *mcr-5.1*. Therefore, the prevalence of *Salmonella* serotypes and their *mcr* variants showed a wide variety in different geographical regions.

Here are some limitations of this study. First, the numerous genomes used in this study were associated with the development of sequencing technology. The genome information used was hard to represent the real prevalence of *mcr*-producing isolates. Second, the majority of the *mcr*-producing *Salmonella* genomes originated from the United States, China, the United Kingdom, and Germany, but data were limited from other countries. Therefore, the above limitations would bias the deduced results.

In conclusion, our study highlighted that *mcr*-producing *Salmonella* isolates have spread in five continents (Asia, Europe, America, Australia, and Africa) globally. Typhimurium and 1,4,[5],12:i:- were the predominant serotypes carrying *mcr* genes in *Salmonella*, and *mcr-9.1* and *mcr-1.1* were the predominant variants. The spread of such various *mcr* variants is of great concern for food safety and public health, and it is urgent to enhance surveillance and control the spread of *mcr* genes among *Salmonella*.

## 4. Materials and Methods

### 4.1. Salmonella Genomes Collected 

We searched two important words, “*mcr*” and “*Salmonella*”, in the public NCBI database on 1 January 2022. A total of 2279 *Salmonella* isolates were positive for *mcr* genes, and the detailed information of isolates, including years, countries, host, and serotypes, were then downloaded. However, 2145 out of 2279 genomes were available because the rest had not been released. These genomes were used for phylogenetic analysis to explore the implied spread of MCR-producing *Salmonella* worldwide.

### 4.2. The Identification of mcr Variants and Serotypes

The genomes used were further to identify the variant types of *mcr* genes through ResFinder (https://cge.cbs.dtu.dk/services/ResFinder/, accessed on 1 June 2022). *Salmonella* serotypes were available from the uploaded information, and the serotypes that were not submitted by the uploader were predicted by SeqSero 1.2 (https://cge.cbs.dtu.dk/services/SeqSero/, accessed on 1 June 2022).

### 4.3. Phylogenetic Analysis

A total of 2145 *Salmonella* genomes from different countries were used for phylogenetic analysis. Single-nucleotide polymorphisms (SNPs) were extracted using Snippy (https://github.com/tseemann/snippy, accessed on 1 June 2022) to generate core genomic alignment. Gubbins [17] was then used to identify and remove recombination regions using an algorithm that iteratively identifies loci containing elevated densities of base substitutions, and then resulting pairwise SNP differences were calculated. The core SNP alignment was used to generate a maximum-likelihood phylogeny using RAxML v8.1.23 (Karlsruhe, Germany) [18] with the GTR nucleotide substitution model. The display, annotation, and management of phylogenetic trees were performed by the ITOL tool [19]. The antibiotic resistance genes in all genomes were identified by ResFinder 4.1 (Lyngby, Denmark) [20].

### 4.4. Data Analysis 

The bar and circle charts used to analyze the prevalence of *mcr* serotypes were generated with GraphPad Prism 7 software (San Diego, California, CA, USA). The world map marked with the distribution of *mcr*-positive *Salmonella* was drawn by Inkscape 0.92 (New York, NY, USA).

## Figures and Tables

**Table 1:** AMR genotypes in *mcr*-producing *Salmonella* isolates.

Antibiotic Resistance Determinants		Numbers	Ratio/%
Carbapenems	*bla*_NDM-1_	1	0.0
Tigecycline	*tet*(X4)	1	0.0
Aminoglycosides	*dfr*A12	232	10.2
-	*dfr*A14	42	1.8
-	*dfr*A17	21	0.9
-	*dfr*A16	59	2.6
-	*dfr*A19	382	16.8
-	*dfr*A23	19	0.8
-	*dfr*A5	35	1.5
-	*dfr*A1	212	9.3
-	*aph*(6)-Id	1023	44.9
-	*aph*(3″)-Ib	1143	50.2
-	*aph*(3′)-Ia	685	30.1
-	*aph*(4)-Ia	246	10.8
-	*aad*A2	931	40.9
-	*aad*A1	1063	46.6
-	*aad*A22	15	0.7
-	*aad*A5	35	1.5
-	*aac*(6′)-IIc	357	15.7
-	*aac*(6′)-Ib-cr5	85	3.7
-	*aac*(3)-IId	129	5.7
-	*aac*(3)-IVa	212	9.3
-	*aac*(3)-IIe	12	0.5
-	*aac*(3)-IIg	160	7.0
β-Lactamase	*bla*_CTX-M-14_	136	6.0
-	*bla*_CTX-M-55_	90	3.9
-	*bla*_CTX-M-65_	14	0.6
-	*bla*_CTX-M-15_	19	0.8
-	*bla*_CTX-M-3_	13	0.6
-	*bla*_CTX-M-9_	109	4.8
-	*bla*_CTX-M-130_	3	0.1
-	*bla*_CTX-M-2_	4	0.2
-	*bla*_CMY-2_	46	2.0
-	*bla*_CMY-16_	1	0.0
-	*bla*_TEM_	1315	57.7
-	*bla*_SHV-12_	307	13.5
-	*bla*_DHA-1_	33	1.4
-	*bla*_OXA-1_	115	5.0
-	*bla*_OXA-10_	3	0.1
Rifampicin	*arr-3*	86	3.8
Chloramphenicol	*cml*A1	292	12.8
-	*cat*B3	76	3.3
-	*cat*A2	296	13.0
Macrolides	*mph*(A)	113	5.0
-	*mef*(B)	7	0.3
-	*erm*(B)	10	0.4
-	*erm*(42)	5	0.2
Fosfomycin	*fos*A7	326	14.3
-	*fos*A3	137	6.0
-	*fos*A4	1	0.0
Florfenicol	*flo*R	480	21.1
Lincomycin	*lnu*(F)	64	2.8
-	*lnu*(G)	8	0.4
Sulfonamide	*sul*3	334	14.7
-	*sul*2	1220	53.5
-	*sul*1	1184	52.0
Tetracyclines	*tet*(A)	578	25.4
-	*tet*(M)	71	3.1
-	*tet*(B)	1099	48.2
-	*tet*(C)	34	1.5
-	*tet*(D)	368	16.1
Quinolones	*qnr*S1	160	7.0
-	*oqx*A	180	7.9
-	*oqx*B	181	7.9
-	*qnr*S2	10	0.4
-	*qnr*B4	32	1.4
-	*qnr*B19	60	2.6
-	*qnr*B2	214	9.4
AMR mutations	*gyrA*_D87G	128	5.6
-	*gyrA*_S83Y	58	2.5
-	*gyrA*_D87N	98	4.3
-	*gyrA*_G81C	1	0.0
-	*gyrA*_S83F	57	2.5
-	*parC*_S80I	12	0.5
-	*parC*_S80R	5	0.2
-	*parE*_S458P	1	0.0

## Data Availability

Not applicable.

## Supplementary Materials

The following supporting information can be downloaded at: https://www.mdpi.com/article/10.3390/antibiotics11080998/s1, Comparison of *mcr* variant sequences from *Salmonella* (.fas profile).
